# 10,12-Conjugated linoleic acid supplementation improves HDL composition and function in mice

**DOI:** 10.1016/j.jlr.2022.100241

**Published:** 2022-06-15

**Authors:** Tomas Vaisar, Shari Wang, Mohamed Omer, Angela D. Irwin, Carl Storey, Chongren Tang, Laura J. den Hartigh

**Affiliations:** 1 University of Washington, Department of Medicine, Division of Metabolism, Endocrinology, and Nutrition, Seattle, WA, USA; 2 University of Washington, Diabetes Institute, Seattle, WA, USA

**Keywords:** HDL proteomics, serum amyloid A, HDL particle size, HDL particle concentration, fast-phase liquid chromatography, cholesterol transporters, alpha-1-antitrypsin, scavenger receptor class B member 1, Abca1, weight loss, Apoa2, apolipoprotein A2, BMDM, bone marrow-derived macrophage, cHDL, control HDL, 10,12 CLA, 10,12-conjugated linoleic acid, CR, caloric restriction, HDL-P, HDL particle, hHDL, human HDL, IMA, ion mobility analysis, Ldlr, LDL receptor, NIH, National Institutes of Health, PA, palmitic acid, PVAT, perivascular adipose tissue, Saa, serum amyloid A, Saa1, serum amyloid A1, Saa2, serum amyloid A2, Serpina1e, alpha-1-antitrypsin 1–5

## Abstract

Obesity is associated with inflammation, insulin resistance, and type 2 diabetes, which are major risk factors for CVD. One dietary component of ruminant animal foods, 10,12-conjugated linoleic acid (10,12 CLA), has been shown to promote weight loss in humans. Previous work has shown that 10,12 CLA is atheroprotective in mice by a mechanism that may be distinct from its weight loss effects, but this exact mechanism is unclear. To investigate this, we evaluated HDL composition and function in obese LDL receptor (Ldlr^−/−^) mice that were losing weight because of 10,12 CLA supplementation or caloric restriction (CR; weight-matched control group) and in an obese control group consuming a high-fat high-sucrose diet. We show that 10,12 CLA-HDL exerted a stronger anti-inflammatory effect than CR- or high-fat high-sucrose-HDL in cultured adipocytes. Furthermore, the 10,12 CLA-HDL particle (HDL-P) concentration was higher, attributed to more medium- and large-sized HDL-Ps. Passive cholesterol efflux capacity of 10,12 CLA-HDL was elevated, as was expression of HDL receptor scavenger receptor class B type 1 in the aortic arch. Murine macrophages treated with 10,12 CLA in vitro exhibited increased expression of cholesterol transporters Abca1 and Abcg1, suggesting increased cholesterol efflux potential of these cells. Finally, proteomics analysis revealed elevated Apoa1 content in 10,12 CLA-HDL-Ps, consistent with a higher particle concentration, and particles were also enriched with alpha-1-antitrypsin, an emerging anti-inflammatory and antiatherosclerotic HDL-associated protein. We conclude that 10,12 CLA may therefore exert its atheroprotective effects by increasing HDL-P concentration, HDL anti-inflammatory potential, and promoting beneficial effects on cholesterol efflux.

With more than two-thirds of US adults characterized as overweight or obese (1), obesity with its associated comorbidities, including CVD and type 2 diabetes, continues to be a major problem. Lifestyle modifications such as exercise and caloric restriction (CR) have proven effective against obesity in the short term, yet obesity persists because of the high incidence for weight regain. Some pharmaceutical approaches to weight loss such as orlistat and GLP-1 receptor agonists have proven efficacy, yet are accompanied by unwanted side effects (2, 3). Bariatric surgery is a sustainable weight loss method that is only indicated for the severely obese (4), leaving people with mild to moderate overweight with few sustainable treatment options. Thus, alternative approaches to achieve long-term weight loss are urgently needed.

The nutraceutical industry has attempted to fill this market gap, with approximately 15% of adults in the US reporting the use of dietary supplements in an effort to lose weight (5). The naturally occurring fatty acid 10,12-conjugated linoleic acid (10,12 CLA) is a major component of widely available CLA weight loss supplements. While the weight loss potential of CLA supplements in healthy subjects is generally low, greater weight loss is achievable in obese populations such as those with features of the metabolic syndrome (6, 7, 8). We have previously shown that male mice deficient in the LDL receptor (Ldlr) that had been rendered obese while consuming a high-fat high-sucrose (HFHS) diet, a model that closely approximates human metabolic syndrome (9), lose significant body weight and fat mass when supplemented with 10,12 CLA for 8 weeks (10). Moreover, these same male mice given 10,12 CLA exhibited lower atherosclerosis levels (11). This effect was particularly striking given that a weight-matched control group undergoing equivalent weight loss because of CR of the HFHS diet did not display improved atherosclerosis, despite a vastly improved metabolic profile including reduced circulating triglycerides, cholesterol, fatty acids, and inflammatory markers (11). Previous studies by others have reported similar antiatherosclerotic effects of 10,12 CLA (12, 13). The mechanism by which 10,12 CLA provides atheroprotection is unclear; however, it may involve improvements in lipid metabolism (14).

HDL-C content is inversely correlated with CVD risk (15), but therapeutics aimed at elevating HDL-C levels failed to confer cardiovascular protection (16, 17). Instead of HDL-C, additional metrics are now considered relevant to the antiatherosclerotic capacity of HDL, including its anti-inflammatory properties and its capacity to promote cholesterol clearance via reverse cholesterol transport (18, 19). In the periphery, HDL plays a critical role in the first step of the reverse cholesterol transport pathway by accepting cholesterol from lipid-laden macrophages through multiple mechanisms, including ATP-binding cassette transporters Abca1 and Abcg1-mediated exchange as well as passive efflux pathways, for subsequent excretion by the liver (reviewed in Ref. (19)), an effect that can now be readily quantified using in vitro assays (20). The anti-inflammatory effects of HDL are mediated by multiple mechanisms including Abca1- and Abcg1-mediated cholesterol efflux from macrophages, which modulate the cholesterol content of the plasma membrane, thus disrupting inflammatory signaling pathways (21). Moreover, the ability of HDL to exert either proinflammatory or anti-inflammatory effects is modulated by its protein cargo, as proinflammatory stimuli and disease states that skew the HDL proteome toward an enrichment in inflammatory proteins lead to decreased anti-inflammatory activity of HDL (22).

In the present study, we hypothesized that dietary 10,12 CLA supplementation mediates improvements in atherosclerosis because of beneficial changes in HDL composition and/or function. Using validated assays in cultured adipocytes and macrophages, we examined various HDL functions in vitro, including the anti-inflammatory potential and cholesterol efflux capacity. We in addition examined HDL particle (HDL-P) concentration and composition using shotgun proteomics analysis. Our findings suggest that these key properties of HDL are altered by 10,12 CLA supplementation, which could contribute to its beneficial effect on atherosclerosis in male mice.

## Materials and methods

### Animal study design

**Fig. 1:**
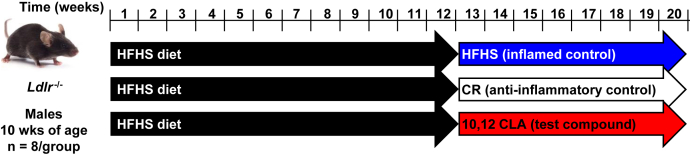
Mouse experimental design. Male *Ldlr*^−/−^ mice (10 weeks of age) were fed an HFHS diet for 12 weeks, then continued on the HFHS diet for an additional 8 weeks with the following: *1*) no variations (mice continued on the HFHS diet); *2*) HFHS diet with 1% added 10,12 CLA; and *3*) HFHS diet plus CR to match the level of weight loss achieved by mice supplemented with 10,12 CLA. Blood was collected for HDL isolation to determine HDL-P number and size distribution, proteomics, cholesterol efflux capacity, and anti-inflammatory capacity. *n* = 8 mice/group.

Details regarding the study design have been published previously (11). Briefly, 10-week-old adult male *Ldlr*^−/−^ mice were randomized into treatment groups and fed an HFHS diet (58.9% kcal from fat [lard], 26.2% kcal from carbohydrates [sucrose] with 0.15% added cholesterol) for 12 weeks (*n* = 8 mice/group). Mice were then switched to one of three test diets for an additional 8 weeks: *1*) HFHS → HFHS diet; *2*) HFHS → HFHS + 1% 10,12 CLA; and *3*) HFHS → HFHS + CR. The study design is shown in Fig. 1. The 10,12 CLA diet replaced 1% of the lard with 1% 10,12 CLA (Nu-Check Prep, Waterville, MN; >90% purity). All test diets were prepared by BioServ (Flemington, NJ) and have been previously described (10). CR begun at 85% total food intake per mouse and adjusted daily to mirror weight loss by 10,12 CLA, ending at an average of 74.4% CR after 8 weeks, as previously described (10). HFHS and 10,12 CLA diets were fed ad libitum, and mice were individually housed for the duration of test diet feeding. Only male mice were used in order to draw direct comparisons with our previous studies (10, 11, 23, 24). At the time of euthanasia, blood was collected and PBS-perfused harvested tissues were snap-frozen in liquid nitrogen and stored at −70°C. All experimental procedures were undertaken with approval from the Institution Animal Care and Use Committee of the University of Washington (#3104-01; March 15, 2013–February 28, 2022) and followed the guidelines of the National Institutes of Health (NIH) guide for the care and use of laboratory animals (NIH Publications No. 8023, revised 1978). We have previously shown that obese male *Ldlr*^−/−^ mice given 10,12 CLA exhibit weight loss because of the loss of white adipose tissue (10). In contrast with mice that experienced equivalent weight loss because of CR, male mice supplemented with 10,12 CLA exhibited less atherosclerosis (11). These phenotypes were replicated in male mice in the current study (data not shown).

### Adipocyte cell culture, HDL isolation, and inflammatory assay

3T3-L1 murine preadipocytes, obtained from American Type Tissue Culture Collection, were propagated and differentiated according to standard procedures (25). HDL was isolated from mouse plasma by ultracentrifugation as described previously (22). Fully differentiated 3T3-L1 adipocytes were pretreated with 50 μg/ml HDL for 6 h in DMEM containing 5 mmol/l glucose and 10% fetal bovine serum and then washed three times with PBS. Adipocytes were then incubated with 250 μM palmitate for 24 h in DMEM containing 5 mmol/l glucose and 10% fetal bovine serum, as described previously (21, 26). HDL isolated from lean chow-fed mice (control HDL [cHDL]) or mice that had been injected with silver nitrate (AgNO_3_, 0.5 ml of a 1% solution injected subcutaneously for 18 h; Sigma) was used as negative and positive controls, respectively. In addition, HDL isolated from plasma from healthy human subjects recruited to the University of Washington Medicine Diabetes Institute was used as an additional negative control (human HDL [hHDL]). All human subjects provided written informed consent and authorization for blood draws and release of medical information (protocol no. 39712, approved by the University of Washington Institutional Review Board in accordance with the Declaration of Helsinki principles).

### RT-PCR

Total RNA from cultured 3T3-L1 adipocytes, macrophages, or mouse tissue was isolated using RNeasy RNA isolation kits (Qiagen), according to the manufacturer’s protocol. About 2 μg total RNA was reverse transcribed into complementary DNA as previously described (11, 25). Expression of genes listed in Table 1 was measured and normalized against *B2m* or *Gapdh* expression, presented relative to media controls (adipocyte and bone marrow-derived macrophage [BMDM] culture) or HFHS-fed mice (tissues), respectively.

**Table 1:** Accession numbers for murine TaqMan primers and sequences for oligonucleotides

Gene name	Thermo Fisher Scientific accession number	
*Gapdh*	Mm99999915_g1	
*B2m*	Mm00437762_m1	
*Saa1*	Mm00656927_g1	
*Saa2*	Mm04208126_mH	
*Saa3*	Mm00441203_m1	
*Ccl2*	Mm00441242_m1	
*Il1b*	Mm00434228_m1	
*Abca1*	Mm00442646_m1	
*Abcg1*	Mm00437390_m1	
*Srb1*	Mm00450234_m1	
Gene name	Forward sequence (5′-3′)	Reverse sequence (5′-3′)
*Gapdh*	AGAACATCATCCCTGCATCC	TCCACCACCCTGTTGCTGTA
*Serpina1e*	GATGGGAAGATGCAGCATC	TCCAGAGATGGACAGTCTG
*Lcat*	GCTTGGGGAATCGGCTAGAA	TGCCGCAGTAAGAAGTGGAG

### J774 macrophage cholesterol efflux assay

Cholesterol efflux capacity of isolated HDL and ApoB-depleted serum (serum-HDL) was quantified using cAMP-stimulated J774 macrophages (American Type Culture Collection) as described previously (20, 27). Serum-HDL was prepared by precipitation of the apoB-containing lipoproteins with PEG 8000 after conversion of plasma to serum by addition of 2.5 mM CaCl_2_. Cells were washed two times with PBS, then incubated in DMEM supplemented with 0.1% (w/v) fatty acid-free albumin, [^3^H]cholesterol (0.5 μCi/ml), and an ACAT inhibitor (34.4 μM; Sandoz) for 24 h at 37°C. After one wash with PBS, the cells were incubated in DMEM + fatty acid-free albumin supplemented with bromoadenosine-cAMP (500 μM; Sandoz) for 24 h. Cells were washed again with PBS and then incubated with HDL (30 μg protein/ml) or serum-HDL (1.5%) for 4 h. Cholesterol efflux capacity (percent of total cholesterol) was determined by the ratio of radiolabeled cholesterol in the medium, corrected for counts in the media without HDL to the sum of both medium and the cell lysates.

### HDL size and concentration determination

HDL-P concentration and size distribution were quantified by calibrated-differential ion mobility analysis (IMA), as described previously (28). Three main HDL subspecies (medium, large, and extralarge) were deconvoluted by curve fitting, and the peak areas for each were quantified using a calibration curve constructed with a protein standard. For total HDL-P concentration, coefficient of variation was <10%, and for the individual subspecies, coefficients of variation were <20%. Fast-phase liquid chromatography was used to separate plasma, as we have done extensively (11, 23, 29, 30). Fractions 30–35, which contain HDL, were used to calculate area under the curve using GraphPad Prism 6 software (GraphPad).

### Proteomics analysis

HDL (d = 1.063–1.21 g/ml) was isolated by sequential density ultracentrifugation from EDTA-plasma (31). Ten micrograms of HDL protein were solubilized with 0.5% sodium deoxycholate (Sigma-Aldrich, St Louis, MO) in 200 mM NH_4_HCO_3_, spiked with 0.5 μg of [^15^N]Apoa1 as internal standard (32), reduced with dithiothreitol, alkylated with iodoacetamide, and digested with two additions of trypsin (1:20, w/w HDL protein; sequencing grade; Promega, Fitchburg, WI) for 4 h, and overnight. After precipitation of sodium deoxycholate with formic acid (1% final concentration), samples were frozen and stored at −20°C until analysis (less than a week). For the LC/MS analysis, an equivalent of 200 ng of HDL protein was injected (33).

### LC-MS/MS analysis

Tryptic digests of HDL (5 μg protein) isolated from obese HFHS-fed mice, HFHS + 10,12 CLA-fed mice, or HFHS + CR-treated mice were analyzed. After desalting on a C18 trapping column (Reprosil-Pur 120 C18-AQ, 5 μm, 0.1 × 40 mm; Dr Maisch HPLC GmbH, Germany) (flow rate of 4 μl/min), the digested peptides were separated on an analytical column (Reprosil-Pur 120 C18-AQ, 5 μm, 250 × 0.075 mm; Dr Maisch HPLC GmbH). Following a multistep linear gradient was used: 1–5% B in 2 min, 5–25% in 50 min, and 25–35% in 10 min. At the end of the gradient, the column was washed with a ramp to 80% B and re-equilibrated (A—0.1% formic acid in water, B—acetonitrile, 0.1% formic acid, and flow rate of 0.4 μl/min). An LC-MS/MS consisting of a nanoAQUITY UPLC (Waters, MA), and a Thermo Q Exactive Plus Orbitrap (Thermo Fisher Scientific, San Jose, CA) mass spectrometer with electrospray ionization were used for the analysis.

### Protein identification

MS/MS spectra were matched against the mouse UniProt database (v. January 2019) using the COMET (v.2018 rev.2) search engine with semitryptic specificity, fixed Cys carbamidomethylation, and variable Met oxidation modifications. The mass tolerance was 20 ppm for both precursor ions and product ions. COMET results were further validated with PeptideProphet and ProteinProphet (34, 35). The following criteria were used to define positively identified proteins: *a*) a high peptide identification score (according to PeptideProphet; *P* > 0.90); *b*) a high protein identification score (*P* > 0.95, ProteinProphet); and *c*) at least two peptides unique to the protein of interest had to be detected in at least four mouse samples. Requiring at least two unique peptides with a high confidence score markedly decreases the false-positive rate of protein identification.

### Immunoblotting

Total protein concentration of HDL preparations or 1% NP-40-lysed BMDMs was determined using the BCA Protein Assay (Thermo Fisher Scientific, Rockford, IL). Immunoblots were performed on equal amounts of protein as described previously (30) and probed for total serum amyloid A (Saa) (R&D Systems; Minneapolis, MN; catalog no.: AF2948), Apoa1 (Rockland Immunochemicals, Inc, Pottstown, PA; catalog no.: 600-101-196), Abca1 (Novus Biologicals, LLC, Littleton, CO; catalog no.: NB400-105), Abcg1 (Novus Biologicals, LLC; Littleton, CO; catalog no.: NB400-132), and actin (Sigma-Aldrich; catalog no.: A5441). Blots were visualized using a LICOR imaging system, and densitometry was performed using ImageJ software (NIH).

### BMDM culture

Bone marrow was isolated from donor C57Bl/6 male mice (*n* = 3) and differentiated into BMDM in RPMI-1640 medium (GE Life Sciences, Pittsburgh, PA) that contained 30% L-cell conditioned medium over the course of 7 days. Nonpolarized BMDMs were treated with media alone (control), 9,11 CLA (100 μM, inert fatty acid control), or 10,12 CLA (100 μM) for 24 h. CLA isomers were conjugated to BSA as described previously (25). An LXR agonist (catalog no.: T0901317; 5 μg/ml; Sigma-Aldrich) was used to augment transporter expression. Total RNA was extracted from >1 × 10^6^ macrophages and reverse transcribed for RT-PCR analysis as described above.

### Statistical analysis

Data were analyzed using GraphPad Prism 6 software and are presented as means ± standard errors. One-way ANOVA was used to compare differences between mice receiving the different diets as indicated, and Bonferroni post hoc testing was used to detect differences among mean values of the groups. A *P* value <0.05 was considered statistically significant.

## Results

### HDL from 10,12 CLA-treated mice is more anti-inflammatory than HDL from HFHS-fed mice

**Fig. 2:**
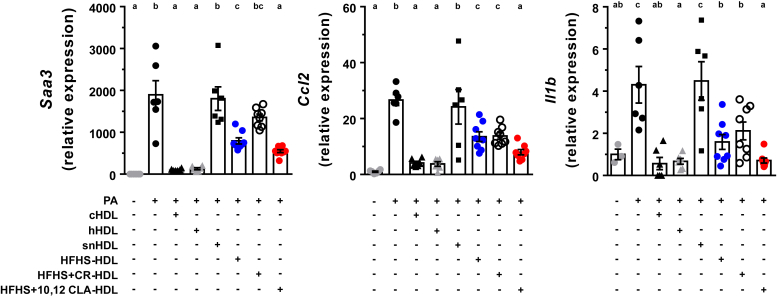
HDL anti-inflammatory assay using cultured adipocytes. Fully differentiated 3T3-L1 adipocytes were pretreated with the indicated mouse and hHDL preparations (50 μg/ml) for 6 h, cells were thoroughly washed three times, then treated with PA (250 μM) for 24 h. Expression levels of genes indicative of inflammation including *Saa3*, *Ccl2*, and *Il1b* were measured and normalized to *B2m*, presented relative to untreated cells (media). Different letters indicate significant differences, assessed using one-way ANOVA with multiple comparisons (Tukey) (*P* < 0.05). cHDL, control HDL from lean chow-fed mice; HFHS + CR-HDL, HDL from mice fed a HFHS diet that were calorically restricted; HFHS-HDL, HDL from mice fed an HFHS diet; HFHS + 10,12-HDL, HDL from mice fed an HFHS diet containing 10,12 CLA; hHDL, human HDL from healthy lean subjects; snHDL, HDL from mice injected with silver nitrate.

It has previously been reported that HDL from lean healthy mice and humans exerts anti-inflammatory activity against palmitic acid (PA)-induced inflammation in adipocytes, an effect that is diminished in HDL from inflamed mice and humans (22). Moreover, anti-inflammatory HDL has been inversely associated with atherosclerotic disease risk (36, 37, 38). To determine if the antiatherosclerotic effect of 10,12 CLA supplementation was coincident with enhanced anti-inflammatory potential of HDL, an HDL inflammatory assay was performed in fully differentiated 3T3-L1 adipocytes. As shown in Fig. 2, cHDL isolated from chow-fed mice and lean human subjects (hHDL), expected to lack atherosclerosis and systemic inflammation, completely blocked gene expression of chemokines serum amyloid A3 (*Saa3*), monocyte chemotactic protein 1 (*Ccl2*), and the cytokine interleukin 1-beta (*Il1b*) induced by PA, whereas HDL from mice that had been injected with inflammatory silver nitrate (AgNO_3_) had no effect, as we have shown previously (22). HDL from HFHS-fed (HFHS-HDL) and HFHS + CR-treated (CR-HDL) male mice partially reduced PA-induced *Saa3* (42 and 72%), *Ccl2* (51 and 52%), and *Il1b* (37 and 49%) gene expression levels, respectively. However, HDL isolated from 10,12 CLA-supplemented male mice (10,12 CLA-HDL) exhibited a larger anti-inflammatory effect than HFHS-HDL and CR-HDL, with adipocyte *Saa3* expression dropping to 29%, *Ccl2* expression to 30%, and *Il1b* expression to 16% of PA-mediated expression levels (Fig. 2). Notably, all HFHS-fed HDL groups had a lower capacity to blunt PA-induced inflammation than cHDL and hHDL, presumably because the HFHS diet promotes low-grade systemic inflammation (9), whereas cHDL and hHDL were isolated from healthy noninflamed donors. Adipocyte treatment with HDL preparations in the absence of PA did not alter inflammatory gene expression (not shown). Thus, HDL from 10,12 CLA-supplemented male mice exhibits a higher anti-inflammatory potential than HDL isolated from obese or calorically restricted mice.

### Medium, large, and total HDL-Ps are increased by 10,12 CLA supplementation

**Fig. 3:**
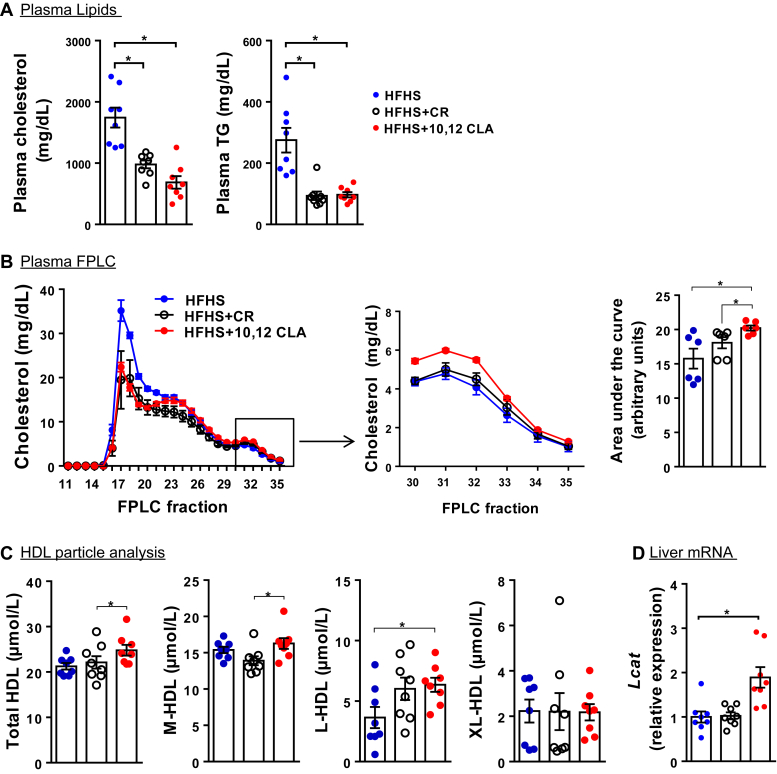
HDL-P size analysis and FPLC. A: Plasma cholesterol and triglycerides. B: Pooled plasma samples from each treatment group were separated by FPLC. Areas under the curve for fractions containing HDL (30, 31, 32, 33, 34, 35) are presented. C: Total, medium (M), large (L), and extralarge (XL) HDL-P concentrations were determined using calibrated-ion mobility analysis. D: Liver *Lcat* mRNA expression, presented normalized to *Gapdh*. *n* = 8 mice/group; ∗*P* < 0.05. FPLC, fast-phase liquid chromatography.

As we have reported previously, mice supplemented with 10,12 CLA exhibit reduced plasma cholesterol and triglyceride levels (Fig. 3A), an effect also observed with weight loss due to CR (11). Fast-phase liquid chromatography fractionation showed elevated HDL levels in mice given 10,12 CLA (Fig. 3B). We therefore used calibrated-IMA to quantify HDL-P_IMA_ (total HDL-P concentration) and the concentrations of three subspecies: medium-HDL (diameter, 9.90 ± 0.07 nm [mean ± SD]), large-HDL-Ps (10.80 ± 0.07 nm), and extralarge-HDL-Ps (12.21 ± 0.10 nm). Median HDL sizes and total HDL-P numbers were consistent with previously reported values in mice (39). In male mice supplemented with 10,12 CLA, total HDL concentrations were 17% and 12% higher than HFHS- and CR-treated mice, respectively (Fig. 3C). Medium-HDL-Ps from 10,12 CLA-treated mice were 6% and 17% higher, whereas large-HDL-Ps were 74% and 6% higher than HFHS- and CR-fed male mice, respectively. There were no differences in extralarge-HDL-P concentration between any groups. Moreover, hepatic expression of *Lcat* was increased by 10,12 CLA (Fig. 3D), suggesting a potential mechanism by which HDL could be remodeled into more mature and larger particles. Thus, supplementation with 10,12 CLA increased the number of HDL-Ps, derived largely from increased medium- and large-sized particles.

### Basal macrophage cholesterol efflux to HDL is higher from mice supplemented with 10,12 CLA than obese or calorically restricted mice

**Fig. 4:**
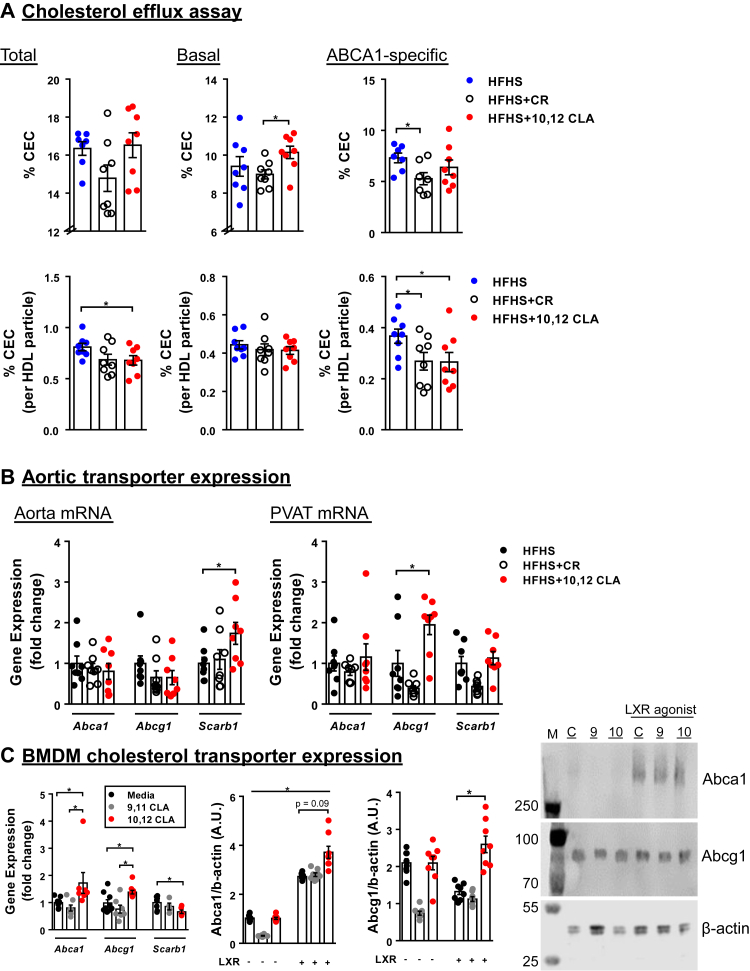
Cholesterol efflux capacity and cholesterol transporter expression. A: Cholesterol efflux capacity (CEC) of the indicated HDL preparations was quantified from radiolabeled cholesterol-loaded J774 macrophages. Total CEC is the sum of basal (Abca1-independent) and Abca1-depenent efflux (determined by pretreatment with a cAMP agonist). B: Cholesterol transporter gene expression from aortic arch and the immediately adjacent PVAT. C: Cholesterol transporter gene (left panel) and protein expression (middle and right panels) from BMDM cultured in the presence or the absence of 100 μM 10,12 CLA or 9,11 CLA (inert control), with or without an LXR agonist (T0901317, 5 μg/ml) for 24 h; *n* = 8 mice/group; ∗*P* < 0.05.

Because cholesterol efflux capacity of HDL is also inversely associated with atherosclerotic disease risk (40), we next examined whether differences in this metric could explain the atheroprotective effect of 10,12 CLA. There was a trend toward elevated total cholesterol efflux capacity from J774 macrophages treated with HDL isolated from mice supplemented with 10,12 CLA (Fig. 4A), driven primarily by increased basal non-cAMP-induced cholesterol efflux rather than Abca1-specific efflux. This effect was abolished when adjusted for particle concentration, with decreased basal and Abca1-mediated efflux from 10,12 CLA-HDL, suggesting that the increased basal efflux capacity of HDL from 10,12 CLA-supplemented mice is driven by increased particle concentration. We next examined expression of genes that are important for cholesterol efflux from macrophages (41). Aortic arch tissue had higher *Scarb1* (scavenger receptor class B type 1) expression from mice given 10,12 CLA (Fig. 4B), one of the possible mediators of HDL cholesterol efflux capacity not induced by cAMP in the in vitro J774 cell efflux system. This suggests that elevated levels of *Scarb1* may increase cholesterol efflux in 10,12 CLA-treated mice. Perivascular adipose tissue (PVAT) immediately adjacent to the aorta exhibited elevated *Abcg1* expression (Fig. 4B). Similarly, BMDMs treated with 10,12 CLA in vitro exhibited increased *Abca1* and *Abcg1* expression, an effect that was not observed with 9,11 CLA, an inert control fatty acid (Fig. 4C). Increased Abca1 and Abcg1 protein expression by 10,12 CLA and an LXR agonist cotreatment was confirmed via immunoblot (Fig. 4C). Collectively, these results suggest that 10,12 CLA treatment may improve both the ability of HDL to accept cholesterol and the ability of the cells to efflux cholesterol.

### Proteomics reveals that 10,12 CLA and CR promote changes in HDL protein distinct from obese mice

**Fig. 5:**
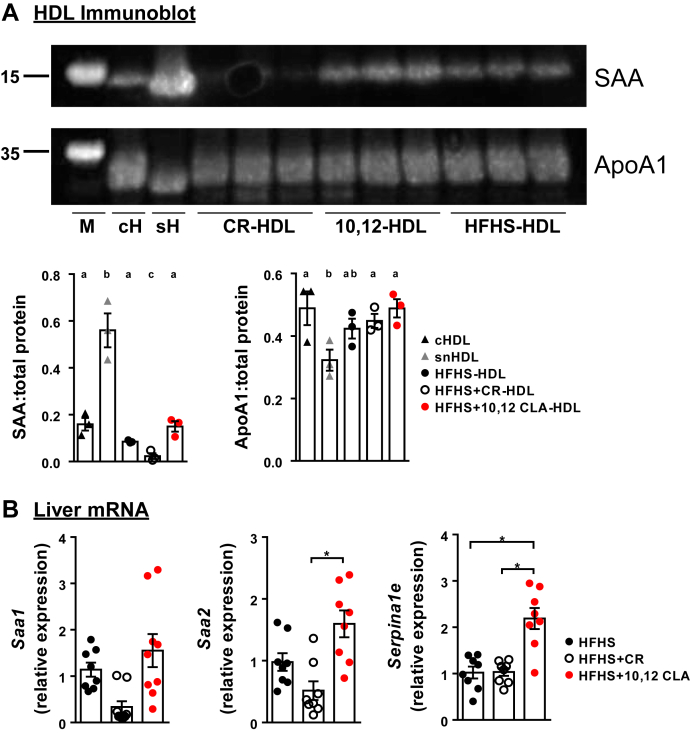
Proteomics confirmation and hepatic gene expression. A: HDL preparations from the indicated treatment groups were subjected to immunoblot and probed for Saa (marker = 15 kD) and Apoa1 (marker = 35 kD). Densitometry was performed using ImageJ software. Control HDL samples were pooled; *n* = 3 for treatment groups. Different letters indicate significant differences, assessed using one-way ANOVA with multiple comparisons (Tukey) (*P* < 0.05). cH, control HDL from mice injected with saline; CR-HDL, HDL from mice fed an HFHS diet that were calorically restricted; 10,12-HDL, HDL from mice fed an HFHS diet containing 10,12 CLA; HFHS-HDL, HDL from mice fed an HFHS diet; sH, HDL from mice injected with silver nitrate. B: Liver gene expression was quantified from the indicated treatment groups. *n* = 8 mice/group, ∗*P* < 0.05 from HFHS.

In order to determine if the protein cargo of HDL differed between groups, isolated HDL was subjected to proteomics analysis. Shotgun proteomics identified 128 proteins in HDL across all samples (supplemental Table S1). Of these, 11 proteins were uniquely altered in CR-HDL and 14 were altered in both CR- and 10,12-HDL when compared with HFHS-HDL, which presumably reflects an effect of weight loss (Table 2). An additional 12 proteins were uniquely altered in 10,12 CLA-HDL, including apolipoprotein A2 (Apoa2), serum amyloid A1 (Saa1), serum amyloid A2 (Saa2), serine protease inhibitor A3K (Serpina3K), prothrombin, and alpha-1-antitrypsin inhibitor 1–5 (Serpina1e) (Table 2). Of these, there are notable differences in Saa1 and Saa2 HDL content, with 28% more Saa1 and 57% more Saa2 in 10,12 CLA-HDL versus HFHS-HDL, and with 30% less Saa1 and 37% less Saa2 in CR-HDL versus HFHS-HDL. Immunoblot of total HDL supports these findings, with notable reductions in Saa HDL content in CR-treated mice and elevated Saa levels in HDL from 10,12 CLA-treated mice (Fig. 5A). No differences in Apoa1, the major HDL protein, were noted between groups. Further, mRNA transcripts of *Saa2* and *Serpina1e* were increased in the liver in 10,12 CLA-treated mice (Fig. 5B). Thus, proteomics analysis has revealed significant differences in the proteome of 10,12 CLA-HDL that may reflect changes in hepatic expression levels.

**Table 2:** Treatment-specific effects on HDL proteomics

Protein name	Gene name	HFHS	HFHS + CR	HFHS + 10,12 CLA
10,12 CLA-specific effects on HDL proteins				
Apolipoprotein A-II^a^	*Apoa2*^a^	179.13^a^	173.63^a^^,^^b^	148.13^a^^,^^b^^,^^c^
Serum albumin^a^	*Alb*^a^	107.38^a^	100.00^a^	85.38^a^^,^^b^
Major urinary protein 18^d^	*Mup18*^d^	25.00^d^	16.25^b^^,^^d^	33.38^b^^,^^c^^,^^d^
Serum amyloid A-1^d^	*Saa1*^d^	15.88^d^	5.88^b^^,^^d^	20.38^c^^,^^d^
Serum amyloid A-2^d^	*Saa2*^d^	10.38^d^	3.13^b^^,^^d^	16.25^b^^,^^c^^,^^d^
Odorant-binding protein 1a^a^	*Obp1a*^a^	6.63^a^	7.38^a^	4.13^a^^,^^c^
Serine protease inhibitor A3K^d^	*Serpina3k*^d^	7.75^d^	6.25^d^	10.75^b^^,^^c^^,^^d^
Prothrombin^a^	*F2*^a^	6.13^a^	4.25^a^	2.63^a^^,^^b^
Secretoglobin family 2B member 2^a^	*Scgb2b2*^a^	4.63^a^	5.00^a^	2.25^a^^,^^c^
Alpha-1-antitrypsin 1-5 (serine protease inhibitor A1e)^d^	*Serpina1e*^d^	1.75^d^	2.00^d^	4.63^b^^,^^c^^,^^d^
Major urinary protein 17^d^	*Mup17*^d^	2.13^d^	1.13^d^	4.38^b^^,^^c^^,^^d^
Major urinary protein 4^a^	*Mup4*^a^	2.25^a^	3.50^a^	0.13^a^^,^^b^^,^^c^
CR-specific effects on HDL proteins				
Serum paraoxonase/arylesterase 1^a^	*Pon1*^a^	122.38^a^	100.88^a^^,^^b^	106.25^a^
Beta-globin^d^	*Hbb-bs*^d^	59.38^d^	86.88^b^^,^^d^	55.38^c^^,^^d^
Flavin reductase (biliverdin reductase B)^d^	*Blvrb*^d^	4.00^d^	8.00^b^^,^^d^	3.50^c^^,^^d^
Transferrin receptor protein 1 (CD antigen CD71)^d^	*Tfrc*^d^	1.63^d^	5.13^b^^,^^d^	3.13^d^
Vitamin D-binding protein (Gc-globulin)^a^	*Gc*^a^	7.13^a^	3.88^a^^,^^b^	4.63^a^
Carbonic anhydrase 2^d^	*Ca2*^d^	0.38^d^	5.13^b^^,^^d^	0.38^c^^,^^d^
Major urinary protein 20^a^	*Mup20*^a^	8.88^a^	4.00^a^^,^^b^	8.00^a^^,^^c^
Carbonic anhydrase 1^d^	*Ca1*^d^	2.13^d^	5.13^b^^,^^d^	1.13^c^^,^^d^
Major urinary protein 3^a^	*Mup3*^a^	6.88^a^	3.63^a^^,^^b^	6.00^a^
Cathepsin D^a^	*Ctsd*^a^	3.50^a^	1.50^a^^,^^b^	2.13^a^
Cytochrome b5^a^	*Cyb5a*^a^	3.88^a^	1.25^a^^,^^b^	2.75^a^
10,12 CLA and CR-specific effects on HDL proteins (weight loss effects)				
Apolipoprotein A-I^d^	*Apoa1*^d^	485.63^d^	530.25^b^^,^^d^	536.13^b^^,^^d^
Apolipoprotein B-100^a^	*Apob*^a^	349.50^a^	249.00^a^^,^^b^	262.50^a^^,^^b^
Apolipoprotein C-III^a^	*Apoc3*^a^	153.25^a^	126.25^a^^,^^b^	126.13^a^^,^^b^
Apolipoprotein E^a^	*Apoe*^a^	133.63^a^	100.75^a^^,^^b^	98.13^a^^,^^b^
Pregnancy zone protein^a^	*Pzp*^a^	40.13^a^	24.75^a^^,^^b^	18.75^a^^,^^b^^,^^c^
Beta-2-microglobulin^a^	*B2m*^a^	24.75^a^	17.13^a^^,^^b^	18.00^a^^,^^b^
N-fatty-acyl-amino acid synthase/hydrolase PM20D1 (peptidase M20 domain-containing protein 1)^a^	*Pm20d1*^a^	20.25^a^	13.25^a^^,^^b^	13.75^a^^,^^b^
Complement factor H^a^	*Cfh*^a^	28.38^a^	12.38^a^^,^^b^	9.38^a^^,^^b^
Predicted gene 20425^a^	*Gm20425*^a^	15.50^a^	4.75^a^^,^^b^	5.00^a^^,^^b^^,^^c^
Complement factor H-related 2^a^	*Cfhr2*^a^	12.50^a^	5.00^a^^,^^b^	4.75^a^^,^^b^^,^^c^
Fibrinogen gamma chain^a^	*Fgg*^a^	6.75^a^	4.13^a^^,^^b^	3.88^a^^,^^b^^,^^c^
Keratin 90^a^	*Krt90*^a^	6.50^a^	3.13^a^^,^^b^	2.50^a^^,^^b^^,^^c^
Beta-2-glycoprotein 1 (apolipoprotein H)^a^	*Apoh*^a^	6.63^a^	3.75^a^^,^^b^	3.25^a^^,^^b^
Plasminogen^a^	*Plg*^a^	11.00^a^	2.25^a^^,^^b^	0.38^a^^,^^b^

HDL proteomics summary of proteins enriched in either 10,12 CLA-supplemented mice, CR-treated mice, or both. Data are presented as mean ± SEM, *n* = 8 mice per group.

a Decreased.

b *P* < 0.05 from HFHS control.

c *P* < 0.05 from CR.

d Increased.

## Discussion

In the present study, we sought to determine if 10,12 CLA supplementation mediates improvements in atherosclerosis via changes in HDL composition and function. Using validated assays in cultured adipocytes and macrophages, we show that HDL isolated from mice that had consumed an HFHS diet containing 10,12 CLA exhibited: *1*) improved anti-inflammatory function in cultured 3T3-L1 adipocytes; *2*) increased HDL-P concentration; and *3*) increased basal cholesterol efflux capacity from J774 macrophages, despite evidence of a proteome that was moderately enriched in proinflammatory mediators such as Saa. Moreover, BMDMs treated with 10,12 CLA had elevated *Abca1* and *Abcg1* expression levels with corresponding elevated *Abcg1* expression levels in PVAT adjacent to atherosclerotic lesions, suggesting that 10,12 CLA may improve the ability of key cells within and around the lesions to efflux cholesterol to HDL, an effect that may be augmented by increased HDL-P concentration.

Previous studies have suggested that 10,12 CLA plays a protective role against atherosclerosis in small animal models (14), but few mechanistic details have been attributed to this antiatherosclerotic effect. Some studies suggest that 10,12 CLA exerts anti-inflammatory effects on cell types associated with atherosclerosis, such as monocytes (42, 43, 44), macrophages (43, 45), smooth muscle cells (46), and endothelial cells (47), with subsequently decreased monocyte/macrophage adhesion potential (48, 49). Perhaps the best described mechanism relates to macrophage polarization. Several studies suggest that 10,12 CLA promotes the polarization of macrophages toward a “resident” or “M2” phenotype (10, 11, 43, 50, 51), which is believed to be atheroprotective (52). M2 macrophages have been shown to have a higher efflux capacity than M1, with much higher Abca1 and Abcg1 expression and protein levels (53). Whether such effects on macrophages directly contribute to reduced atherosclerosis levels remains to be determined.

In addition to these anti-inflammatory effects, 10,12 CLA has been reported to have diverse effects on cholesterol metabolism. Animal supplementation studies by us and others suggest a cholesterol-lowering effect of 10,12 CLA (11, 54), an effect that is absent in most human CLA supplementation studies (55). Moreover, 10,12 CLA has also been shown to increase HDL levels (56, 57), an effect also observed in the present study. However, whether 10,12 CLA improves HDL function was unclear, providing the rationale for the present study. The current study supports previous evidence that 10,12 CLA supplementation increases HDL levels and expands this finding to indicate 10,12 CLA-mediated increases in medium- and large-HDL-P numbers, which could contribute to increased basal cholesterol efflux capacity. A few previous studies suggested that 10,12 CLA increases Abca1 expression from macrophages with concurrent increased cholesterol efflux capacity (58, 59), whereas other studies showed that 10,12 CLA had no effect on cholesterol efflux (60, 61). We now show that 10,12 CLA treatment increases *Abcg1* expression from the PVAT surrounding atherosclerotic aortas and increases both *Abca1* and *Abcg1* expression in BMDM. Mice doubly deficient in Abca1 and Abcg1 exhibit worsened atherosclerosis (62). Thus, increased cholesterol transporter expression, when synergized with the increased number of total, medium, and large HDL-Ps, may contribute to increased Abca1-mediated and basal cholesterol efflux capacity of HDL from 10,12 CLA-treated mice.

A previous study has presented HDL proteomics data from *Apoe*^−/−^ mice supplemented with 1% 10,12 CLA (57). The results of this study are not in agreement with ours, instead showing that levels of Apoa1 decreased, whereas Apoa2 and Apoc3 increased in response to 10,12 CLA. By contrast, in our study, we found *increased* Apoa1 and *decreased* Apoa2 and Apoc3 associated with HDL. The reasons for opposing results between that study and ours are not immediately clear but likely relate to the different models utilized (*Apoe*^−/−^ vs. *Ldlr*^−/−^), which promote vastly different hyperlipidemic phenotypes with variable levels of VLDL and possibly plasma levels of Apoe and Apoc3. However, these changes in apolipoproteins are not likely to drive the atheroprotection by 10,12 CLA, as CR-treated mice exhibited the same apolipoprotein changes but not the atheroprotection. Thus, these changes in apolipoprotein HDL content are likely driven by weight loss, as has previously been reported in humans undergoing intermittent fasting-driven weight loss (63).

Notably, while both CR- and 10,12 CLA-HDL had higher levels of Apoa1 (presumed to be an effect of weight loss), 10,12 CLA-HDL had higher levels of Saa1 and Saa2. This was unexpected given that the presence of Saa subtypes on HDL has been suggested to displace Apoa1 during an acute phase response (64), yet this did not appear to be the case in the present study because of the increase in *both* Apoa1 and Saa1/2. Likely the small but significant increase in Saa1/2 is insufficient to alter HDL function as much as larger increases associated with acute inflammation. It is also possible that it was overshadowed by the larger increase in the more abundant Apoa1, contributing to a net increase in HDL-P concentration and in cholesterol efflux capacity and atheroprotection.

A notable proteomic difference between 10,12 CLA-HDL and CR-HDL was Serpina1e, which was enriched on 10,12 CLA-HDL. Serpina1e is a liver-derived circulating protease inhibitor that may play an important role in anti-inflammation (65). Individuals that exhibit Serpina1e insufficiency often lack control over inflammatory responses, and direct introduction of Serpina1e to peripheral blood mononuclear cells reduces inflammation (66, 67). Interestingly, Serpina1e is also produced by M2 macrophages (68). While Serpina1e deficiency has been well documented to associate with chronic obstructive pulmonary disease, Serpina1e is now also recognized as a cardioprotective protein (69), whereby genetic defects in Serpina1e promote atherosclerotic CVD (70) and atherosclerosis is inversely proportional to Serpina1e levels in rabbit models (71). There is some evidence that Serpina1e may play a role in aortic lesion stabilization in mice (72). Moreover, HDL-associated Serpina1e is linked with atheroprotection by exhibiting an anti-inflammatory effect on J774 macrophages (73), antielastase activity, and enhanced cholesterol efflux capacity of small dense HDL-Ps (74, 75). Thus, it is also plausible that the increased Serpina1e on HDL from 10,12 CLA-supplemented mice also offsets the increased proinflammatory Saa1/2 and enhances cholesterol efflux capacity to promote atheroprotection.

While we now show that key structural and functional changes to HDL provide a potential mechanism for 10,12 CLA-induced atheroprotection, there are some limitations in our study design. In our previous studies, we have utilized 9,11 CLA as a control group but have not observed any notable effects on body weight and energetics (10, 23), atherosclerosis (11), cellular metabolism (25), or gut microbiota phenotypes (24). Thus, we did not include 9,11 CLA as a control group in this study but instead included a calorically restricted weight loss control group. It is possible that 9,11 CLA induces similar changes to HDL, which in turn may not impact atherosclerosis (76), suggesting a more complex mechanism by which 10,12 CLA exerts atheroprotection. Indeed, supplementation with mixed CLA (an equal ratio of the 9,11 and 10,12 CLA isomers) has been reported to increase HDL levels in mice (56), rats (77), and humans (78), although HDL function was not assessed in these prior studies. Another limitation is that HDL functionality was only assessed ex vivo. It is therefore unclear whether the small changes in HDL-P number, composition, and efflux potential would lead to notable changes in whole-body cholesterol homeostasis and atherosclerosis. Future studies could examine reverse cholesterol transport using radioactive tracers in live animals. Finally, our observation that liver *Lcat* expression is increased by 10,12 CLA, which could be a potential mechanism by which 10,12 CLA promotes changes in HDL-P dynamics, raises additional questions, as there have been conflicting studies reporting both beneficial and detrimental effects of LCAT on atherosclerosis in mice (79).

In summary, we report herein a potential mechanism by which 10,12 CLA-induced weight loss is atheroprotective in male mice (11). We present evidence that such mice have more HDL-Ps in the medium to large range that exert a stronger anti-inflammatory and passive cholesterol efflux phenotype than obese mice as well as weight-matched control mice. Proteomics analysis revealed that while HDL from 10,12 CLA-supplemented mice exhibited elevated Apoa1 and Serpina1e protein levels, it also contained higher levels of Saa1 and Saa2 than control mice. However, the improved anti-inflammatory and cholesterol efflux outcomes in the 10,12 CLA group suggest that this increased proinflammatory HDL cargo plays a negligible role in HDL function. We conclude that improved HDL function, assessed in a multifaceted approach, could be an important mechanism of atheroprotection in mice losing weight because of 10,12 CLA supplementation.

## Data availability

All data described are contained within this article and/or in supplemental data.

## Supplemental data

This article contains supplemental data.

## Conflict of interest

The authors declare that they have no conflicts of interest with the contents of this article.
