# Supplementary material for: 10,12-Conjugated linoleic acid supplementation improves HDL composition and function in mice
Source: J Lipid Res. 2022 Jun 15;63(8):100241. doi: 10.1016/j.jlr.2022.100241 (PMC9283942; doi:10.1016/j.jlr.2022.100241)
Supplement: Supplemental Table S1 [file mmc1.docx]

**Supplemental Table S1.**

| **Protein name** | **Gene name** | **HFHS-HDL** | **HFHS+CR-HDL** | **HFHS+10,12 CLA-HDL** |
| --- | --- | --- | --- | --- |
| **Apolipoprotein A-I** | ***Apoa1*** | **485.63** | **530.25*** | **536.13*** |
| **Apolipoprotein B-100** | ***Apob*** | **349.50** | **249.00*** | **262.50*** |
| **Apolipoprotein A-II** | ***Apoa2*** | **179.13** | **173.63*** | **148.13*^#^** |
| **Apolipoprotein C-III** | ***Apoc3*** | **153.25** | **126.25*** | **126.13*** |
| **Serum paraoxonase/arylesterase 1** | ***Pon1*** | **122.38** | **100.88*** | **106.25** |
| **Apolipoprotein E** | ***Apoe*** | **133.63** | **100.75*** | **98.13*** |
| **Serum albumin** | ***Alb*** | **107.38** | **100.00** | **85.38*** |
| **Beta-globin** | ***Hbb-bs*** | **59.38** | **86.88*** | **55.38^#^** |
| H-2 class I histocompatibility antigen, Q10 alpha chain | *H2-Q10* | 74.75 | 59.00 | 62.13 |
| Serum amyloid A-4 protein | *Saa4* | 70.88 | 52.63 | 68.50 |
| Apolipoprotein A-IV | *Apoa4* | 50.75 | 56.00 | 48.63 |
| Apolipoprotein D | *Apod* | 49.13 | 46.50 | 43.75 |
| Apolipoprotein C-II | *Apoc2* | 43.63 | 43.25 | 38.00 |
| Apolipoprotein C-I | *Apoc1* | 48.00 | 40.25 | 40.38 |
| Complement C3 | *C3* | 36.25 | 36.50 | 34.63 |
| Hemoglobin subunit alpha | *Hba* | 28.25 | 37.38 | 25.38 |
| Phospholipid transfer protein | *Pltp* | 31.25 | 30.25 | 29.38 |
| Prosaposin | *Psap* | 34.25 | 29.38 | 29.88 |
| Apolipoprotein N | *Apon* | 34.63 | 24.13 | 28.88 |
| Complement C4-B | *C4b* | 24.25 | 23.88 | 22.38 |
| Murinoglobulin-1 (MuG1) | *Mug1* | 29.63 | 29.88 | 25.00 |
| Clusterin (Apolipoprotein J) | *Clu* | 23.63 | 21.75 | 22.63 |
| Apolipoprotein M | *Apom* | 20.88 | 22.38 | 21.50 |
| Alpha-1-antitrypsin 1-1 (Serine protease inhibitor A1a) | *Serpina1a* | 27.38 | 23.38 | 25.25 |
| **Pregnancy zone protein** | ***Pzp*** | **40.13** | **24.75*** | **18.75*^#^** |
| Fibrinogen alpha chain | *Fga* | 23.25 | 19.13 | 19.25 |
| **Beta-2-microglobulin** | ***B2m*** | **24.75** | **17.13*** | **18.00*** |
| Phosphatidylcholine-sterol acyltransferase (Lecithin-cholesterol acyltransferase) | *Lcat* | 18.50 | 15.88 | 15.75 |
| Napsin-A | *Napsa* | 17.38 | 14.13 | 13.50 |
| **N-fatty-acyl-amino acid synthase/hydrolase PM20D1 (Peptidase M20 domain-containing protein 1)** | ***Pm20d1*** | **20.25** | **13.25*** | **13.75*** |
| **Major urinary protein 18** | ***Mup18*** | **25.00** | **16.25*** | **33.38*^#^** |
| Prenylcysteine oxidase | *Pcyox1* | 16.38 | 11.63 | 12.00 |
| BPI fold-containing family A member 2 | *Bpifa2* | 10.88 | 10.50 | 13.63 |
| H-2 class I histocompatibility antigen, L-D alpha chain | *H2-L* | 17.63 | 12.75 | 13.13 |
| Insulin-like growth factor-binding protein complex acid labile subunit (ALS) | *Igfals* | 4.88 | 9.50 | 8.75 |
| **Complement factor H** | ***Cfh*** | **28.38** | **12.38*** | **9.38*** |
| Anthrax toxin receptor 2 | *Antxr2* | 8.63 | 9.75 | 7.75 |
| Predicted gene 8909 | *Gm8909* | 14.50 | 11.38 | 10.63 |
| Alpha-2-HS-glycoprotein | *Ahsg* | 10.63 | 9.38 | 9.50 |
| **Serum amyloid A-1 protein** | ***Saa1*** | **15.88** | **5.88*** | **20.38^#^** |
| Keratin, type I cytoskeletal 10 | *Krt10* | 8.63 | 8.50 | 7.13 |
| **Predicted gene 20425** | ***Gm20425*** | **15.50** | **4.75*** | **5.00*^#^** |
| Retinol-binding protein 4 | *Rbp4* | 10.00 | 9.38 | 8.75 |
| Apolipoprotein C-IV | *Apoc4* | 10.13 | 9.50 | 7.88 |
| Predicted gene 7298 | *Gm7298* | 10.25 | 9.25 | 9.13 |
| **Immunoglobulin heavy constant mu** | ***Ighm*** | **10.88** | **8.38** | **4.38*^#^** |
| **Flavin reductase (Biliverdin reductase B)** | ***Blvrb*** | **4.00** | **8.00*** | **3.50^#^** |
| **Transferrin receptor protein 1 (CD antigen CD71)** | ***Tfrc*** | **1.63** | **5.13*** | **3.13** |
| Cystatin E/M | *Cst6* | 7.38 | 7.25 | 8.00 |
| **Serum amyloid A-2** | ***Saa2*** | **10.38** | **3.13*** | **16.25*^#^** |
| Inter alpha-trypsin inhibitor, heavy chain 4 | *Itih4* | 8.38 | 6.50 | 6.38 |
| Phosphatidylinositol-glycan-specific phospholipase D (Glycosyl-phosphatidylinositol-specific phospholipase D) | *Gpld1* | 6.25 | 5.88 | 5.13 |
| Vitronectin | *Vtn* | 7.50 | 6.13 | 5.88 |
| Predicted pseudogene 5478 | *Gm5478* | 6.00 | 6.00 | 3.50 |
| **Odorant-binding protein 1a** | ***Obp1a*** | **6.63** | **7.38** | **4.13^#^** |
| Fibrinogen beta chain | *Fgb* | 6.88 | 6.63 | 5.38 |
| Uncharacterized protein C5orf46 homolog | *Gm94* | 5.38 | 5.38 | 4.88 |
| Keratin, type II cytoskeletal 5 | *Krt5* | 4.88 | 5.13 | 2.50 |
| Keratin, type II cytoskeletal 1 | *Krt1* | 5.13 | 5.88 | 4.63 |
| Predicted gene, 49369 | *Gm49369* | 5.88 | 3.75 | 4.75 |
| Pantetheinase (Vascular non-inflammatory molecule 1) | *Vnn1* | 4.13 | 5.50 | 2.88 |
| CD97 antigen | *Cd97* | 1.88 | 3.50 | 3.13 |
| Tissue factor pathway inhibitor | *Tfpi* | 5.88 | 5.50 | 5.13 |
| Serum paraoxonase/lactonase 3 | *Pon3* | 6.13 | 3.75 | 3.88 |
| **Serine protease inhibitor A3K** | ***Serpina3k*** | **7.75** | **6.25** | **10.75*^#^** |
| Indian hedgehog protein | *Ihh* | 6.25 | 4.25 | 5.00 |
| **Vitamin D-binding protein (Gc-globulin)** | ***Gc*** | **7.13** | **3.88*** | **4.63** |
| Alpha-1-antitrypsin 1-2 (Serine protease inhibitor A1b) | *Serpina1b* | 5.50 | 5.63 | 5.13 |
| Vascular cell adhesion protein 1 | *Vcam1* | 5.13 | 5.88 | 4.75 |
| Predicted gene 5938 | *Gm5938* | 5.00 | 5.50 | 3.13 |
| **Complement factor H-related 2** | ***Cfhr2*** | **12.50** | **5.00*** | **4.75*^#^** |
| **Fibrinogen gamma chain** | ***Fgg*** | **6.75** | **4.13*** | **3.88*^#^** |
| Transthyretin | *Ttr* | 5.13 | 5.00 | 4.63 |
| **Keratin 90** | ***Krt90*** | **6.50** | **3.13*** | **2.50*^#^** |
| Histocompatibility 2, Q region locus 4 | *H2-Q4* | 4.88 | 4.00 | 4.00 |
| Alpha-1-antitrypsin 1-4 (Serine protease inhibitor A1d) | *Serpina1d* | 5.25 | 5.00 | 3.38 |
| **Prothrombin** | ***F2*** | **6.13** | **4.25** | **2.63*** |
| L-selectin | *Sell* | 4.13 | 3.88 | 3.88 |
| **Carbonic anhydrase 2** | ***Ca2*** | **0.38** | **5.13*** | **0.38^#^** |
| **Major urinary protein 20** | ***Mup20*** | **8.88** | **4.00*** | **8.00^#^** |
| **Carbonic anhydrase 1** | ***Ca1*** | **2.13** | **5.13*** | **1.13^#^** |
| **Angiopoietin-related protein 3** | ***Angptl3*** | **5.25** | **5.50** | **2.38*^#^** |
| **Beta-2-glycoprotein 1 (Apolipoprotein H)** | ***Apoh*** | **6.63** | **3.75*** | **3.25*** |
| Immunoglobulin kappa constant | *Igkc* | 4.38 | 3.63 | 2.13 |
| Peroxiredoxin-2 | *Prdx2* | 2.13 | 4.00 | 1.63 |
| Keratin, type II cytoskeletal 6A | *Krt6a* | 3.38 | 1.75 | 1.38 |
| **Secretoglobin family 2B member 2** | ***Scgb2b2*** | **4.63** | **5.00** | **2.25^#^** |
| Integrin beta-2 (CD antigen CD18) | *Itgb2* | 2.75 | 1.63 | 2.75 |
| ER membrane protein complex subunit 9 | *Emc9* | 3.25 | 3.00 | 3.13 |
| **Major urinary protein 3** | ***Mup3*** | **6.88** | **3.63*** | **6.00** |
| Glutathione peroxidase 3 | *Gpx3* | 2.75 | 4.13 | 2.75 |
| **Cathepsin D** | ***Ctsd*** | **3.50** | **1.50*** | **2.13** |
| Actin, cytoplasmic 1 (Beta-actin) | *Actb* | 2.00 | 1.50 | 0.75 |
| Profilin-1 (Profilin I) | *Pfn1* | 3.00 | 4.75 | 3.50 |
| Platelet-activating factor acetylhydrolase | *Pla2g7* | 3.13 | 3.00 | 2.25 |
| Glycolipid transfer protein domain-containing protein 2 | *Gltpd2* | 1.63 | 1.63 | 1.75 |
| Keratin 78 | *Krt78* | 2.25 | 2.63 | 1.63 |
| Keratin, type I cytoskeletal 19 | *Krt19* | 3.00 | 2.00 | 1.75 |
| **Plasminogen** | ***Plg*** | **11.00** | **2.25*** | **0.38*** |
| Keratin, type I cytoskeletal 14 | *Krt14* | 2.63 | 1.25 | 0.75 |
| Importin-5 | *Ipo5* | 1.25 | 1.88 | 0.88 |
| Intercellular adhesion molecule 1 | *Icam1* | 2.00 | 2.75 | 2.75 |
| Sulfhydryl oxidase 1 (Quiescin Q6) | *Qsox1* | 1.25 | 1.38 | 1.63 |
| RIKEN cDNA 2210010C04 gene | *2210010C04Rik* | 2.13 | 2.00 | 2.63 |
| Peptidyl-prolyl cis-trans isomerase C | *Ppic* | 3.13 | 1.50 | 1.75 |
| Pulmonary surfactant-associated protein B (SP-B) | *Sftpb* | 2.00 | 2.13 | 1.00 |
| **Cytochrome b5** | ***Cyb5a*** | **3.88** | **1.25*** | **2.75** |
| Keratin, type I cytoskeletal 13 | *Krt13* | 2.13 | 2.25 | 1.63 |
| Ras-related protein Rap-1A | *Rap1a* | 1.88 | 3.00 | 2.63 |
| Lymphocyte antigen 6E | *Ly6e* | 2.88 | 2.13 | 2.00 |
| Protein LEG1 homolog | *Leg1* | 4.25 | 4.38 | 2.88 |
| Keratin, type I cytoskeletal 42 | *Krt42* | 2.00 | 1.25 | 1.00 |
| Ras-related protein Rab-11B | *Rab11b* | 2.13 | 1.25 | 2.88 |
| MHC classIb T15 | *Gm11127* | 3.13 | 1.63 | 1.63 |
| Arylsulfatase G | *Arsg* | 1.75 | 1.75 | 1.50 |
| ADP-ribosylation factor 3 | *Arf3* | 1.13 | 1.25 | 2.38 |
| Coagulation factor XIII A chain | *F13a1* | 1.88 | 1.50 | 1.50 |
| Platelet factor 4 | *Pf4* | 1.88 | 1.88 | 1.38 |
| Protein AMBP | *Ambp* | 1.75 | 1.50 | 1.25 |
| Cathelicidin antimicrobial peptide | *Camp* | 1.88 | 2.13 | 1.13 |
| **Alpha-1-antitrypsin 1-5 (Serine protease inhibitor A1e)** | ***Serpina1e*** | **1.75** | **2.00** | **4.63*^#^** |
| Lipase | *Lipo1* | 2.50 | 3.50 | 1.63 |
| Parvalbumin alpha | *Pvalb* | 1.63 | 1.38 | 1.50 |
| Lipase | *Lipo2* | 2.00 | 2.38 | 1.50 |
| Thrombospondin-1 | *Thbs1* | 1.50 | 2.63 | 1.25 |
| **Major urinary protein 17** | ***Mup17*** | **2.13** | **1.13** | **4.38*^#^** |
| Secretoglobin, family 2B, member 7 | *Scgb2b7* | 1.88 | 2.63 | 1.63 |
| **Major urinary protein 4** | ***Mup4*** | **2.25** | **3.50** | **0.13*^#^** |
| ABPBG18 (Secretoglobin, family 2B, member 18) | *Scgb2b18* | 2.00 | 2.00 | 1.38 |

*p<0.05 from HFHS

^#^p<0.05 from 10,12 CLA
